# NANOmetric BIO-Banked MSC-Derived Exosome (NANOBIOME) as a Novel Approach to Regenerative Medicine

**DOI:** 10.3390/jcm7100357

**Published:** 2018-10-15

**Authors:** Bruna Codispoti, Massimo Marrelli, Francesco Paduano, Marco Tatullo

**Affiliations:** 1 Tecnologica Research Institute, 88900 Crotone, Italy; bruna.codispoti@tecnologicasrl.com (B.C.); francesco.paduano@tecnologicasrl.com (F.P.); 2 Marrelli Health, 88900 Crotone, Italy; prof.marrelli@libero.it

**Keywords:** MSCs, EVs, exosomes, tissue regeneration, immunomodulation, biobanking

## Abstract

Mesenchymal stem cells (MSCs) are well known for their great potential in clinical applications. In fact, MSCs can differentiate into several cell lineages and show paracrine behavior by releasing endogenous factors that stimulate tissue repair and modulate local immune response. Each MSC type is affected by specific biobanking issues—technical issues as well as regulatory and ethical concerns—thus making it quite tricky to safely and commonly use MSC banking for swift regenerative applications. Extracellular vesicles (EVs) include a group of 150–1000 nm vesicles that are released by budding from the plasma membrane into biological fluids and/or in the culture medium from varied and heterogenic cell types. EVs consist of various vesicle types that are defined with different nomenclature such as exosomes, shedding vesicles, nanoparticles, microvesicles and apoptotic bodies. Ectosomes, micro- and nanoparticles generally refer to the direct release of single vesicles from the plasma membrane. While many studies describe exosomes as deriving from multivesicular bodies, solid evidence about the origin of EVs is often lacking. Extracellular vesicles represent an important portion of the cell secretome. Their numerous properties can be used for diagnostic, prognostic, and therapeutic uses, so EVs are considered to be innovative and smart theranostic tools. The aim of this review is to investigate the usefulness of exosomes as carriers of the whole information panel characterizing the use of MSCs in regenerative medicine. Our purpose is to make a step forward in the development of the NANOmetric BIO-banked MSC-derived Exosome (NANOBIOME).

## 1. Introduction

Mesenchymal stem cells (MSCs) are almost ubiquitous in the adult body. Initially this population of immature cells has only been isolated in bone marrow, but further studies have described their presence in a wide range of organs (hearth, brain) tissues (fat, oral cavity), and liquid fluids (blood, urine, semen) [1,2].

MSCs are characterized by their elevated proliferation ability. Furthermore, their great plasticity confers them the potential to differentiate into mature specialized cells with a great variety of phenotypes (bone, cartilage, fat, neuron) [3,4], regardless of the original source of isolation.

The discovery that MSCs do not express HLA (human leukocyte antigen) class II has shed light on the low immunogenicity of these cells in allogeneic transplantation [5]. Further studies have illustrated that these cells are able to modulate the immunologic response and inflammation in an HLA-independent fashion [6].

Thus, the regenerative and immunomodulatory characteristics of MSCs have been exploited for the treatment of a multitude of clinical situations including the regeneration of traumatic injuries, autoimmune disease, cancer, neurologic disorders, and heart illnesses. More than 800 clinical trials have been approved for the most disparate clinical pathological conditions (clinicaltrials.gov) based on MSC therapy. 

Although stem cell banks are widespread throughout the world, offering available samples to be transplanted as needed, MSC biobanking encounters many technical and regulatory issues linked to the risks associated with the use of cells in clinics.

The intense paracrine activity of MSCs has been strongly explored, and consists of cytokine and chemokine production, mitochondrial transfer, and extracellular vesicle secretion [7]. 

Extracellular vesicles are small double-lipid layer particles that are secreted by budding of the plasma membrane and released into the extracellular space. These nano- and micro-vesicles (such as exosomes, apoptotic bodies, nanoparticles, and microvesicles) carry proteins, mRNA, and miRNA among neighboring cells conveying a multitude of information, and stimuli. Many studies have focused on EVs investigation, mainly because these small structures embed the secretome of their originating cell. EVs secreted by MSCs have been studied in different model diseases in order to elucidate their great paracrine power for the regeneration of injured tissues and their significant immunomodulation ability.

EVs can be isolated from liquid fluids or harvested from a conditioned MSCs medium. Some investigations have tested the stability of EVs stored under different conditions as alternatives for cell biobanking. These should overcome some regulatory problems without altering the effectiveness of the induced biological functions [8].

Clinical trials are ongoing, with the aim of confirming the diagnostic and therapeutic potential of EVs. Many attempts must be made to shed light on the ideal stabilizer and cryoprotectant agent to be used and on the correct temperature degree to ensure effective long-lasting stability of stored EVs. The creation of a systematic process for EV biobanking could represent a very advantageous system for disposing of the powerful properties of MSC secretome, avoiding the typical issues linked to cell-therapy, and finally, moving towards the new frontiers of exosome-based therapy and diagnostics. 

## 2. MSC Biobanking: Ethical, Technical, and Regulatory Issues

Biobanks for stem cell storage are growing in number in order to satisfy the increasing needs for available immature populations for research and clinical use.

Embryonic stem cells, in the early blastocyst stage, are able to differentiate in all organs and tissues of the body (totipotency); fetal stem cells can differentiate into tissues of different germ layers (pluripotency). Despite the important potential for clinical application, many ethical concerns limit the use of these cells from human sources [9]. Moreover, another limit of these highly immature populations is the real risk of insurgence of teratomas [10].

Likewise, stem cells residing in the adult body have a differentiation potential limited to a relatively finite number of specialized tissues (multipotency). Their isolation and application bypass the ethical matters linked with embryonic and fetal stem cell sources [11].

Adult stem cells are unable to give rise to teratomas, but some evidence indicates the advent of secondary solid tumors after hematopoietic stem cell transplantations from bone marrow [12,13]. The United States of America agency—Food and Drugs Administration (FDA) established basal concerns for stem cell manipulation, including the definition of sample identification, in order to evaluate the autologous or unrelated setting. The absence of extraneous substances is potentially capable of altering cell behavior and efficacy. The absence of microorganism contamination is necessary to avoid disease transmission. Furthermore, the sample should be successfully efficient to promote the required biological effects [14]. 

In addition to this, many issues arise from the definition of stem cell use as being a common “practice of medicine” or as a “biological drug”; in the latter case, procedures must be suitable for FDA approval.

Despite some clinicians asserting that stem cell products are not drugs and that they should not be regulated by the Food and Drug Administration, FDA regulations argue that these clinics generally produce stem cell products that can be classified as biological drugs. The approval for production and clinical use of stem cell biological drugs implies acceptance by the FDA of an Investigational New Drug (IND) application that requires a Biological License Application (BLA) (http://www.ipscell.com/2015/01/stemhumanexperiment).

Furthermore, isolation of MSCs from different sources often does not allow an adequate clinical number of cells to be directly transplanted [15]; hence, the passage of in vitro expansion is frequently required. This handling step is in contrast with the FDA key concept of “minimal manipulation” that distinguishes clinical products from biological drugs that, by definition, require “more than minimal manipulation” processes [16]. Another central concept for the FDA is the “homologous use”, that is, the application of stem cells derived from a specific tissue source, to transplantation in the same tissue of isolation, for example, blood-derived MSCs can only be used for hematologic applications (http://www.ipscell.com/2015/01/stemhumanexperiment). 

The most commonly employed sources of MSCs for biobanking are adipose tissue and umbilical cord tissues, probably due to the easy accessibility and reduced invasiveness required for cell collection. Each cell source requires specific protocols and guidance approval for safe and high quality isolation, manipulation, cryopreservation and storage [14]. During the medical banking of MSC, particular consideration should be given to the process of cryopreservation, for example, the reproduction of perfect temperature variation during freeze and thaw passages, durable storage in pharmaceutical-grade liquid nitrogen, the optimal selection of a cell storage container, the freezing device used and the adequate and approved composition of cryopreservation media [17].

## 3. Extracellular Vesicles

**Figure 1:**
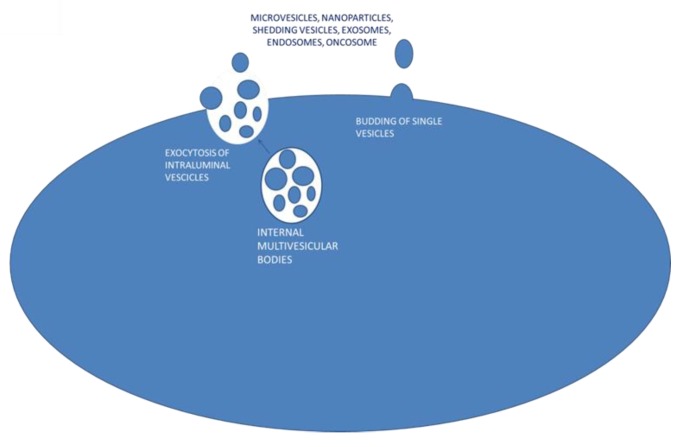
Proposed mechanisms for extracellular vesicle secretion.

Extracellular vesicles (EVs) are secreted by virtually all cell types and have been found in vivo in biologic fluids [18] including blood, urine, saliva, breast milk, ascites, cerebrospinal fluid, and semen [19]. EVs from the cell membrane are present in a wide size range; they can be large (1 μm), a few micrometers long, or up to about 150 nanometers long [20]. Based on their dimensions, EVs are defined as microvesicles (MVs) or nanovesicles. Other authors use different definitions such as micro and nanoparticles, shedding vesicles, apoptotic bodies, exosomes, endosomes or names derived from the cell of origin, such as oncosomes, which are secreted by tumor cells. The mechanism of EV formation includes endosomal formation and maturation by internal budding in endosomal space of multiple vesicles called intraluminal vesicle (ILV). The late-formed endosomes turn into a multivesicular body (MVB) that fuses with the plasma membrane (PM) and releases their internal vesicle content as exosomes by a mechanism of exocytosis [21]. Alternatively, EVs could be directly released to the extracellular space by outward budding from the plasma membrane surface [22] (Figure 1). Many authors define exosomes as the EVs secreted from MVBs while single vesicle budding refers to microvesicles. These definitions are yet to be collectively accepted.

### 3.1. Exosomes: The Good, the Bad and the Ugly

In the 1980s, exosomes were described as vesicles of endosomal origin with sizes ranging from 30 to 1000 nm. In 1987, the term “exosome” was first used to describe small membrane vesicles formed by the vesiculation of intracellular endosomes and released by exocytosis [23].

These vesicles are released during reticulocyte differentiation as a consequence of the fusion of multivesicular bodies (MVBs) with the plasma membrane [24,25]. At the time of their discovery, exosomes were thought to be “storage bodies” for the cell’s unwanted components. In 1996, Raposo et al. demonstrated, by electron microscopy (EM), the presence of secreted exosomes containing MHC class II molecules, previously labeled by BSA gold tracer, in Epstein–Barr virus (EBV)-transformed B cell lines. Furthermore, the protein composition of the secreted exosomes was shown to differ from that of the plasma membrane. These findings suggested the potential endocytic origin of exosomes and led to new interesting hypotheses on the exosome function [26]. 

In the 1990s, various studies on blood cells proposed the involvement of the endosome in central cellular processes, such as blood coagulation, intercellular communication, lipid metabolism [27,28], in the release of inflammatory mediators [29] and in the proliferation of endothelial and immunologic cells [30].

The discovery of tumor-derived extracellular vesicles attracted scientific interest in the involvement of extracellular vesicles in suppressing the immune system against a tumor [31,32], but also in activating the immunologic anti-tumor response [33]. Moreover, tumor exosomes have been shown to participate in cancer angiogenesis [34] and in the spreading of metastatic cells [35]. 

These interesting findings prompted those studying EVs to establish a database named *Vesiclepedia* that includes data on mammalian exosomes (http://microvesicles.org) [36] and also another database including studies of non-mammalian EVs (http://evpedia.info) [37]. Both databases are continuously updated, making them an important instrument to improve EV knowledge.

### 3.2. Exosomes: Individuation and Isolation Strategies

The initial attempts to purify exosomes basically consisted of differential centrifugation, in which successive centrifugation steps are performed at increasing speeds, allowing the recovery of fractions with decreasing dimensions, cells, dead cells, and cellular debris. Then, a final ultracentrifugation at 100,000× *g* allows exosome recovery, followed by washing for the removal of protein aggregates [38]. Alternative protocols have been used to replace the differential centrifugation steps with a single filtration step, such as the use of 0.22 µm filters, an increase in the ultracentrifugation speed to 140,000× *g* [39], or size exclusion chromatography to recuperate units larger than 50,000 kDa, permitting the segregation of soluble proteins [40]. The inclusion of an extra purification step using a sucrose gradient has been used to determine the sedimentation of protein aggregates through sucrose, while vesicles float into a specific position within the sucrose gradient. These methods allow the aggregates of proteins to be separated from membrane-enclosed vesicles that could be available for therapeutic use [41]. Recently, many commercially available kits have been produced by companies for EV isolation that ensure quick and easy purification protocols exploiting immune labeling with magnetic beads, the use of specific filters, and polymer-based precipitation. These procedures, moreover, allow the direct recovery of protein and/or nucleic acids carried by exosomes. The choice of the best method for EV isolation is related to the source of exosomes (i.e., biological fluid specimen, cell supernatant) and the type of analysis to be performed, such as observation, enumeration, flow cytometer investigation, proteomic studies, or RNA isolation. Due to the 200 nm resolution limit of classical optical microscopes, EVs are mainly observed by electron microscopy (EM)—the election technique to observe the small sizes and morphologies of exosomes [26]. Nanoparticle tracking analysis (NTA) is a device that is capable of statistically calculating the diameter of laser-illuminated individual particles by tracking their movement under Brownian motion [42], allowing the evaluation of the size and distribution of EVs. Fluorescent labeling of vesicles using lipid dyes allows the identification of aggregates and large size EVs by fluorescent microscopy [43]. The last generation flow cytometers are able to identify microparticles in the forward scatter channel, but with scarce discrimination efficacy [44].

### 3.3. Exosomes: Composition and Contents

Exosomes are composed of an external lipid bilayer that is mainly enriched in saturated fatty acids, sphingomyelin, phosphatidylserine, cholesterol, and ceramides [45]. Interestingly, the exposition of phosphatidylserine on the surface membrane of exosomes which, in live cells, is confined to the inner leaflet of the PM, has been used to characterize these membrane vesicles by binding with annexin V [46].

Another component of exosomes is cytoplasmic and transmembrane proteins. The protein pattern is often the same as that of the deriving cell. Specifically, this is the case for proteins from the cytosol and the plasma membrane; however, peptides typical of other cellular organelles are absent, thus sustaining the endosomal origin of EVs. Some specific protein subsets are shared by exosomes derived from heterogeneous cell types [47]. In 2007, Valadi et al. detected the presence of mouse proteins in human cells fed with mouse exosomes. These peptides were shown to be absent in exosomes; therefore, it has been hypothesized that exosomes carry the related genetic information. These results demonstrate that exosomes also carry mRNA that is able to be translated into proteins [48]. In 2008, miRNAs were identified in mixed EVs derived from glioblastoma and blood cells [49,50]. Recent NSG techniques elucidated the presence of ulterior genetic material embedded in EVs, including noncoding RNA, with potential regulatory effects on the genomes of target cells [51]. Recent research has demonstrated changes in the composition of EVs after environment alteration mimicking pathological conditions. These changes concern alterations in the protein and RNA content [52,53], as well as changes in the lipid composition [54] of the EV membrane. 

Despite the numerous model disease-based experimental works that have demonstrated that the functional effects of MSC exosomes are carried by their RNA content, other works have suggested that MSC exosomes most probably work through the protein rather than the RNA, due to an inadequate RNA configuration and/or concentration. However, for this to be true, proteins would need to be present in MSC exosomes in a representative therapeutic dose capable of producing a biologically relevant response, especially for the catalytic activity of enzymes [55]. Further investigation is needed to better understand the roles and related effects of the different components of EVs.

### 3.4. Exosomes: Biogenesis and Secretion

The formation of exosomes follows an endocytic process that consists of the internalization of extracellular elements. The formed early endosome starts a maturation process consisting of the internal budding of intra lumen vesicles. These late endosomes are referred to as multi-vesicular bodies. The MVBs could be addressed through lysosomal degradation or, to a lesser extent, by exocytosis [56]. A possible mechanism of intra lumen vesicle formation involves the endosomal sorting complex required for transport (ESCRT), and a multiprotein machinery organized into four subunits (ESCRT 0, 1, 2 and 3) that is associated with supportive proteins (VPS4, VTA1, ALIX also called PDCD6IP) [57]. Interestingly, the inactivation of the entire ESCRT complex does not limit MBV formation; thus, further mechanisms should be concomitantly active [58]. These alternative methods may involve the tetraspanin CD63 [59], or the lipid metabolism enzymes neutral sphingomyelinase [60], and phospholipase D2 [61].

Multiple mechanisms are involved in EV secretion. For example, elevation of the intracellular calcium levels has been documented to induce secretion [62]. Tumor cells spontaneously secrete exosomes (oncosomes) with invasive properties [43], but changes in extracellular conditions could also trigger EV secretion. The interaction between dendritic cells and CD4^+^ T lymphocytes has been demonstrated to induce exosome secretion [63]. The release of neurotransmitters stimulates oligodendroglial exosome secretion that is internalized by neurons through endocytosis [64].

The budding of intraluminal vesicles implies interactions with the cytoskeleton and binding with PM or other membranous compartments that are mediated by a family of GTPases named Rab proteins [65]. This is followed by a SNARE–SNAPs protein interaction [66] that mediates membrane fusion and exosome release. Many other molecules are involved in biogenesis and in intra- and extracellular EV secretion, and the related cellular processes remain a matter of debate.

The limited dimensions of EVs makes their tracking difficult after budding in the extracellular space. To exert their functions on target cells, exosomes must be captured and internalized. The specific binding could be mediated by ligands and receptors present in both EVs and recipient cells. The blocking of the integrins tetraspondin, ICAM-1 and LFA-1 with specific antibodies partially inhibits the interaction of dendritic cell-derived EVs with recipient dendritic cells [67], and heparan sulphate proteoglycans expressed by tumor-EVs are recognized by tumor cells [68]. The final step is the transfer of EV content into the recipient cell. This passage could be done through either an endocytic or phagocytic method, followed by the degradation of; the internalized vesicles to extract their components. The contents of exosomes could also be directly released into the cytosol. This is the case for nucleic acids that influence the gene expression of target cells, for instance, the fusion of EVs with the plasma membrane or with the endocytic membrane must occur. Abrami et al. demonstrated that after endocytosis of the anthrax lethal toxin, these components were found in the intraluminal space of MVBs and in the cytosol of target cells. Moreover, they could be delivered to the extracellular medium as exosomes and infect neighboring cells. These results functionally demonstrate membrane fusion [69,70]. 

## 4. Exosomes from MSCs

The secretory ability of MSCs has been well established by enumerable scientific reports; thus, the identification of exosomes as carriers of stimuli and information among cells suggests a central role of EVs as effectors of the paracrine activity of MSCs (Table 1).

As previously explained, exosomes mainly exert the same features as the cells they came from, such as the transport of proteins, miRNA, mRNA, and other soluble factors implied by MSCs’ functions, including immunomodulation and tissue regeneration [70].

Early evidence describing the implication of exosomes in MSCs’ paracrine activity was illustrated in a model of acute kidney injury. The authors demonstrated that microvesicles derived from human bone marrow MSCs stimulate proliferation and induce resistance to apoptosis in tubular epithelial cells. The administration of MSC microvesicles into SCID (severe combined immunodeficiency) mice accelerates functional recovery after glycerol-induced acute kidney injury. Moreover, the administration of RNAse abolishes the described in vitro and in vivo regenerative effects. These authors concluded that the RNA content of the administrated MSC microvesicles activates proliferation and injury recovery [71]. 

It is important to note that RNAse acts only in conjunction with detergent to lyse the cholesterol-rich phospholipid membrane of vesicles. This shell, in fact, guarantees the protection of exosome content from extracellular insults during passages among cells.

Another study described the exosome-induced recovery effects in a mouse model of myocardial ischemia/reperfusion injury. In this work, EVs of 50- to 100-nm sized particles secreted from MSCs were visualized by electron microscopy and purified by size exclusion fractionation on a HPLC. The administration of the isolated exosomes was shown to reduce the infarcted area in mice [72]. The properties of MSC-derived exosomes have been further explored in several disease models; some investigations focused on the composition and morphology of MSC exosomes.

Exosomes secreted from MSCs express typical mesenchymal markers such as CD105, CD29, CD90, CD44, and CD73, together with more exosome-specific surface antigens including CD107, CD63, CD9, and CD81 [73].

The proteomic profile studies performed by using mass spectrometry and antibody array revealed the different protein compositions in different HPLC-purified MSC-derived exosome preparations with only 20% correspondence in all three batches. The same study identified the presence of a proteasome subunit in MSC exosomes. These findings not only suggest proteasome as a direct actor in contrasting diseases, but also indicate that the proteasome activity could explain the changes in exosome composition during a cell’s life span [74]. Other studies investigated the miRNA contents of MSC-derived exosomes. Tian Sheng Chen et al. suggested that MSCs could enable miRNA-mediated communication among cells by secreting microvesicles rich in miRNA. Microarray analysis and Q-RT PCR results showed that these miRNAs encapsulated in MSC-derived microvesicles were predominantly present as pre-miRNA and thus, in their precursor form [75].

### 4.1. Exosomes from MSCs: Regenerative Potential

The regenerative potential of exosomes derived from MSCs has been investigated in various model diseases and in many different works referred to kidney, liver, heart, and neural injuries.

EVs secreted from mesenchymal stem cells carry several active molecules that have been implicated in central cell regenerative mechanisms including the prevention of cell apoptosis, the promotion of cell proliferation, and the improvement of neovascularization [76,77].

In a study of neurotoxicity induced by smoke and HIV, the neuroprotective potential of exosomes derived from astrocytes carrying antioxidants was proposed to protect neurons against oxidative damage [78]. Another study revealed the presence of neural growth factor transcripts in exosomes from adipose derived-MSCs through verification by immunohistochemical techniques and footprint analysis. These EVs were able to promote neurite outgrowth in vitro and increase regeneration in vivo after sciatic nerve injury [79]. Research on Alzheimer’s disease has shown that exosomes secreted by NCS have a promoting role in beta-amyloid production and clearance; hence, exosomes have been proposed as a target in Alzheimer’s therapy [80].

In an interesting work which challenged the notion that ROS have an exclusively nerve degenerative function, axonal regeneration and functional recovery after spinal injury were demonstrated to be induced by NOX2 (nitric oxide) carried by macrophage-secreted exosomes. Thus, NOX2 may induce the oxidation and inactivation of PTEN (Phosphatase and tensin homolog), leading to PI3K (Phosphatidyl-Inositol 3-Kinase)-phosphorylated (p-) protein kinase *B* signaling and activation of regenerative progress [81].

MSC exosomes have been shown to promote skeletal muscle regeneration by inducing miRNA-mediated myogenesis and angiogenesis in vitro and muscle regeneration in a mouse model of muscle injury [82].

Mesenchymal stromal cell-derived fractionated secretomes enriched in exosomes enhance recovery in in-vitro and in vivo liver injury models [83].

A resident population of MSCs found within the renal glomeruli was shown to contribute to the stimulation of ischemia-reperfusion-related acute kidney injury (AKI) recovery in SCID mice through the release of EVs [84]. RNAse treatment decreased the described regenerative effects. A down-regulation of miRNA production induced by Drosha knockdown contracted the intrinsic regenerative potential of mesenchymal stromal cell-derived EVs in a mouse model of glycerol-induced acute kidney injury [85]. This evidence suggests the central role of RNA in MSC-EV-mediated AKI recovery.

Intramyocardial injection of EVs released by MSCs enhanced the in vitro proliferation, migration, and tube formation of endothelial cells, improved blood flow recovery, and reduced infarct size in an acute myocardial infarction rat model [76]. Exosomes isolated from cord blood-derived, Akt-overexpressing MSCs promoted angiogenesis and lesion size reduction in a rat model of acute myocardial infarction by angiogenesis activation mediated by *PDGF-D* that was up-regulated in the Akt exosomes [86]. Exosomes derived from MSCs overexpressing *GATA-4* improved survival and reduced apoptosis in a culture of rat neonatal cardiomyocytes, as well as restoring cardiac contractile function, and reducing infarct size in vivo. These effects have been attributed to the enhanced expression of miR-19a that down-regulates PTEN, thereby activating the Akt and ERK signaling pathways [87].

Recently, researchers have focused their studies on human embryonic stem cell-derived MSCs (ESC-MSCs). Specifically, the components contained in their conditioned medium were carefully analyzed by multidimensional protein identification technology, cytokine antibody array, gene microarray, and quantitative RT-PCR assays. Computational analysis of the obtained data predicted the presence of gene products involved in metabolism, the immunological response, and in tissue differentiation, including neo-angiogenesis, hematopoiesis, and bone formation [88]. As previously described, exosomes derived from ESC-MSCs showed the ability to restore cardiac functionality after myocardial ischemia/reperfusion injury and severe infarction [72,74]. Furthermore, exosomes were reported to have a key role in promoting miRNA-mediated cell-to-cell communication [75]. 

Despite the clear demonstration of the effects of MSC-derived exosomes in several clinical conditions, the induction of pluripotent stem (iPS) cell-derived exosomes represents a novel field of interest.

A research team investigated exosomes obtained from human embryonic stem cell-induced mesenchymal stem cells (ESC-MSCs) and used them in a mouse model affected by the destabilization of the medial meniscus. Their results showed a significant reduction of the induced osteoarthritis mediated by ESC-MSCs, exerted through regulation of the cartilage synthesis/degradation process [89].

Zhang et al. proposed a ‘cell-free’ therapeutic approach for the treatment of osteochondral defects, demonstrating the ability of human embryonic MSC-derived exosomes to repair cartilage and subchondral bone injuries [90]. An interesting paper by Lai RC et al. proposed the large-scale production of exosomes from human ESC-MSCs after their C-Myc-mediated immortalization, challenging the conventional concept of pharmaceutical manufacturing and introducing the role of MSCs as “producers” of therapeutics [91].

Some authors investigated the regenerative potential of exosomes secreted by iPSC-derived MSCs. Such exosomes were shown to promote neovascularization after excision of the femoral artery in a mouse model of hind-limb ischemia, thus demonstrating a protective effect on limb ischemic injury [92].

In a rat model of a skin wound, exosomes released by human-induced pluripotent stem cell-derived MSCs were shown to be capable of stimulating cutaneous wound healing through the promotion of collagen production and by triggering angiogenesis [93]. The intravenous injection of iPS-MSC-derived exosomes into a steroid-induced osteonecrosis of the femoral head rat model importantly limited bone degradation. Finally, in vitro experiments on iPS-MSC-derived exosomes showed an increase in the migration of endothelial cells and proliferation mediated by PI3K/Akt signaling pathway activation [94].

### 4.2. Exosomes from MSCs: Immunomodulation

The ability to regulate the immune system is a central feature of MSCs and is mainly exerted by their important paracrine activity which involves the release and transport of bioactive molecules that could be mediated by extracellular vesicle secretion.

Exosomes secreted by MSCs exert direct effects on immune cells. The addition of MSC-EVs in a culture of peripheral blood mononucleated cells reduced the growth and differentiation of B cells and decreased the production of IgM, IgG, and IgA [95]. In a culture of splenic mononuclear cells from a mouse model of autoimmune encephalomyelitis, exosomes from MSCs were shown to counteract auto-reactive lymphocyte expansion, induce apoptosis of activated T cells, and initiate Treg production and the secretion of IL-10 and TGF-β anti-inflammatory cytokines [96]. Additionally, in monocytes/macrophages, the administration of MSCs-EVs was shown to increase the levels of IL-10 anti-inflammatory cytokines and reduce the secretion of pro-inflammatory interleukins 1β, 6, 12p40, and TNF-α [97]. Furthermore, different works have described the induction of the M1 to M2 phenotypic transition of macrophages treated with EVs [98,99].

The immunomodulatory effects of MSC-EVs have been investigated in various disease models. Exosomes secreted by MSCs up-regulate IL-10 and TGF-β1 in peripheral blood mononuclear cells isolated from asthmatic patients, thus stimulating the proliferation and immune-suppression capacity of regulatory T lymphocytes. These effects led to the alleviation of inflammation in asthma disease [100].

Exosomes derived from human umbilical cord MSCs were shown to restore organ function in a model of carbon tetrachloride (CCl_4_)-induced fibrotic liver by reducing collagen deposition and decreasing (TGF)-β1 and Smad2 expression, leading to an inhibition of epithelial-to-mesenchymal transition, thus opposing inflammation and fibrosis [101].

In a mouse model of renal ischemia/reperfusion injury, macrophage activation was suppressed by exosomes from MSCs that express high levels of CLR2 (Cryptic loci regulator 2). CLR2-expressing exosomes were shown to be capable of binding free CCl_2_ and abolishing their activity in macrophage recruitment, thereby down-regulating inflammation [102]. Liu et al. demonstrated that exosomes from bone marrow-derived MSCs possess strong pro-angiogenic properties, reduce neuronal cell apoptosis, stimulate axonal regeneration, suppress inflammation, and attenuate lesion size after traumatic spinal cord injury (SCI) [103]. Exosomes derived from human cord blood derived-MSCs increase the functional recovery after SCI through the down-regulation of inflammatory cytokines, including IL-6, TNF-α, IFN-γ, and MIP-1α [104]. 

An interesting report highlighted the role of a particular class of EVs derived from apoptotic bodies, which they call ApoEVs, that is able to modulate the immune system in both activating and suppressing ways [105]. ApoEVs strongly interact with antigen-presenting cells by direct or cross-presentation mechanisms and promote the clearance of apoptotic cells by recruiting phagocytic cells. Related effects may include the production of autoantibodies, consequently favoring autoimmune conditions such as systemic lupus erythematous [106] although the reduced clearance of dying cells has been described as a promoter of autoimmunity [107]. Furthermore, ApoEVs could stimulate antitumor immunity, with exosomes from melanoma cells facilitating the passage of tumor antigens to antigen-presenting cells [108]. The same mechanisms provide a protective effect in infective conditions. ApoEVs secreted by tuberculosis-infected macrophages carry microbial-derived antigens to antigen presentation cells that recruit CD4 and CD8 T lymphocytes [109].

## 5. Biobanking of Exosomes

As described above, exosomes are acquiring even more importance in the clinic due to their central roles as diagnostic and prognostic biomarkers [110], therapeutic targets [111], and drug vehicles [112].

**Figure 2:**
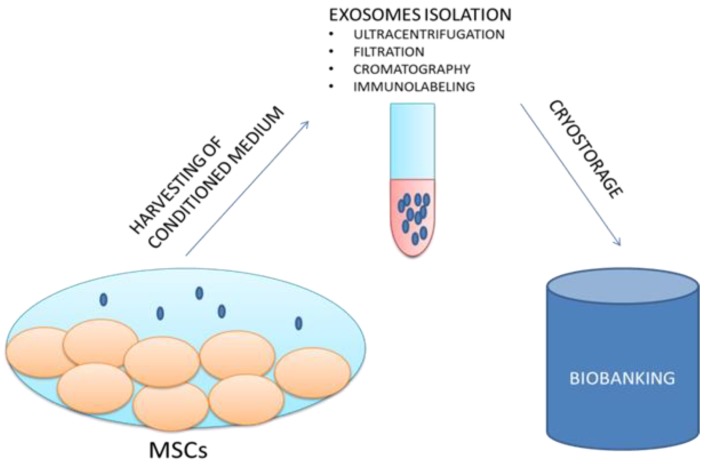
The proposed NANOBIOME approach.

The possibility of storing exosomes in biobanks could represent an advantageous system for various medical applications as well as for research purposes (Figure 2). 

“NANOBIOME” is the acronym of NANOmetric BIO-banked MSCs-derived Exosome, a novel approach that is based on the biobanking of exosomes secreted by MSCs as opposed to managing batches of MSCs. Furthermore, the biological role of exosomes as the holder of the secretome and as the external carrier of the functional biological effects of their generating cells confers to exosomes a significant potential role for innovative “cell-free” regenerative medicine.

Biobanks can currently be defined as facilities where high-quality biological samples are acquired, processed, and preserved by long-term storage for clinical distribution or for future research investigations [113].

Each sample collected and stored in a biobank has to be inserted in a database containing precise information about demographic and clinical data [114].

Liquid fluids (peripheral blood, serum, plasma, urine, saliva, semen), solid tissues, cells (isolated peripheral blood mononuclear cells (PBMCs), stem cells, and other cell types), and biomolecules (RNA, and DNA) are only some of the variety of sample types that are processed and stored in biomedical and research biobanks.

The increasing significance of exosome use in cancer, metabolic diseases, traumatic injuries, and other complex diseases has made this biocomponent amenable for biobanking, and it has great potential advantages for the diagnosis and treatment of these particular diseases [115].

Exosomes exert the ability to cross biological barriers and to selectively reach target cells and organs due to their intrinsic homing ability. Moreover, they are well tolerated by the human body with proper circulation times, and finally, their membranes can be modified to express or incorporate specific molecules. All of these benefits could be exploited for drug delivery [116].

The biobanking process requires the formulation of defined protocols for the collection, sampling, and storage of samples. 

The reduced size of exosomes limits their morphologic observation with conventional optical microscopes; thus, their identification has been mainly assessed by flow cytometry, electron microscopy, and NTA methods. Furthermore, EV harvesting may require different centrifugation techniques associated or not with the filtration and chromatography steps. Immunolabeling is instead exploited by numerous commercially available kits specific for the isolation of exosomes from different biological sources.

Despite much scientific evidence reporting procedures for EV identification and isolation, less information concerning the methods needed for cryopreservation and the storage stability of exosomes is available. Different procedures are required depending on the source of isolation [8].

Kalra et al. tested three different methods for the isolation of exosomes from human plasma, with better results being obtained for the OptiPrepTM density gradient method. Furthermore, they investigated the stability of harvested exosomes for 90 days at different storage temperatures, with superior results being shown for cryopreservation at −80 °C [117]. The stability of exosomes derived from HEK 293T, ECFC, and MSCs cell types was observed during storage at 37 °C, 4 °C, and −20 °C. The initial particle size of 110 nm was assessed by scanning SEM and dynamic light scattering NTA analyses. Storage at 4 °C and 37 °C was shown to induce a size decline and degradation of exosomes, while repeated freeze cycles at −20°C and thawing did not alter the behavior of exosomes [118].

In an ISEV (International Society of Extracellular Vesicles) position paper that summarizes discussions that took place at the ISEV research workshop in New York in 2012, the recommendations for the storage of EVs include the use of siliconized vessels to prevent the adherence of EVs to batch surfaces. Furthermore, the suggested resuspension medium is PBS and the best storage temperature is considered to be −80 °C. Interestingly, neither the freeze and thawing cycles, nor the osmolarity environment, seem to affect the stability of EVs [119].

An investigation on EVs derived from neutrophilic granulocytes confirmed a better storage stability at −80 °C for 4 weeks. In contrast, EVs reduced in size and number at −20 °C and 4 °C, respectively [120].

The basic nature of biobanking requires the study of protocol standardization, including precise evaluation of samples in terms of the harvesting rate, characterization, pre-clinical parameter assessment, factors that could affect the long-term storage of samples, database archiving, and distribution. In addition, complete information regarding the life cycle of the exosomes is critical for the understanding of sample integrity and quality.

More investigations are needed to bridge this gap of knowledge and to create standardized GMP (Good-Manufacturing Practice) protocols for safe and effective exosome cryopreservation and storage, in order to obtain optimal quality samples for applications in the most disparate clinical situations.

## 6. Conclusions

The almost infinite power of the MSC secretome is currently well known, as demonstrated by the growing number of clinical applications involving these cells. The discovery that extracellular vesicles budding from MSCs conserve and spread precious biological information, allows many of the regulatory issues linked to cell therapy to be overcome.

The previously proposed novel “NANOBIOME” approach is based on the biobanking of EVs secreted by MSCs for their easy and available storage and distribution. The standardization of protocols for the isolation and cryopreservation of MSC exosomes could represent a very attractive and useful topic for regenerative medicine purposes.

## Figures and Tables

**Table 1:** Mesenchymal stem cells (MSC)-derived extracellular vesicles (EVs).

EVs Definition	Tissue of Origin/Exosome Source	Content/Composition	Functions	References
Microvesicles	Human bone marrow MSCs	mRNA	Protection against acute tubular injury	[71]
Exosomes	Human ESC-derived mesenchymal stem cell		Decreases myocardial ischemia/reperfusion injury	[72]
Exosomes	Human ESC-derived mesenchymal stem cells	20S proteasome	Reduce the accumulation of misfolded proteins in a mouse model of myocardial infarction	[74]
Microparticles	Human embryonic stem cell (hESC)-derived MSC	pre-miRNA	Enable miRNA-mediated intercellular communication	[75]
Extracellular vesicles	Human bone marrow mesenchymal stem cells		Stimulate neoangiogenesis, preserve cardiac function in a rat myocardial infarction model	[76]
Exosomes	Human umbilical cord mesenchymal stem cells		Improves recovery in cisplatin-induced acute kidney injury rat model	[77]
Exosomes	Rat Adipose-Derived Mesenchymal Stem Cells	Neural growth factors transcripts	Increase neurite outgrowth in vitro and enhance regeneration after sciatic nerve injury in vivo	[79]
Exosomes	Macrophages	Functional NADPH oxidase 2 complexes	Axonal regeneration through a NOX2-PI3K-p-Akt signalling pathway	[81]
Exosomes	Human bone-marrow-derived mesenchymal stem cells	Repair-related miRNAs, miR-494	Stimulation of myogenesis and angiogenesis in vitro, and muscle regeneration in an in vivo model of muscle injury	[82]
Exosomes	Rat bone marrow MSCs	Exosome enriched mesenchymal stromal cell-derived fractionated secretome	Repair and healing of injured liver tissue	[83]
Extracellular vesicles	MSCs within the glomeruli (Gl-MSCs)	miRNAs	Recovery of in a mouse model	[84]
Extracellular vesicles	Bone Marrow MSCs	microRNA	microRNA depletion in EVs from MSCs decrease their intrinsic regenerative potential in acute kidney injury	[85]
Exosomes	Akt-Modified Human Umbilical Cord Mesenchymal Stem Cells	Platelet-derived growth factor D (PDGF-D)	Improve angiogenesis and promote cardiac regeneration	[86]
Exosomes	GATA-4 overexpressing mesenchymal stem cells	Anti-apoptotic microRNAs, miR-19a	Cardio-protection	[87]
Exosomes	Human embryonic stem cell-induced mesenchymal stem cells		Relieve osteoarthritis through the regulation of synthesis/degradation of cartilage extracellular matrix	[89]
Extracellular vesicles/exosomes	Human embryonic mesenchymal stem cells		Stimulation of osteochondral regeneration and repair	[90]
Exosomes	Human induced pluripotent stem cells-derived MSCs		Alleviate hind-limb ischemia and stimulate angiogenesis in mice	[92]
Exosomes	Human induced pluripotent stem cells-derived MSCs		Stimulation of angiogenesis and collagen synthesis accelerating cutaneous wound healing in rats	[93]
Exosomes	Human induced pluripotent stem cells-derived MSCs		Increasing in endothelial cells migration and proliferation and reduction of bone degradation in an osteonecrosis of the femoral head rat model	[94]
Membrane vesicles	Bone marrow human MSCs		Immunosuppressive effect on B lymphocytes	[95]
Microvesicles	Murine bone-marrow derived MSCs	PD-L1, galecin-1 and membrane-bound TGF-β	Initiation of peripheral tolerance and regulation of immune responses	[96]
Exosomes	Human embryonic stem cell (ESC)-derived MSCs		Prolonging of the survival of allogenic skin graft in mice and increased Tregs.	[97]
Extracellular vesicles	Human adipose derived-MSCs		Exert anti-Inflammatory effects, stimulate macrophages switching from a M1 to a M2 phenotype	[98]
Extracellular vesicles	Porcine adipose tissue-derived MSCs	Anti-inflammatory cytokine interleukin (IL) 10	Reduction of renal inflammation, increasing of medullary oxygenation in porcine model of metabolic syndrome and renal artery stenosis	[99]
Exosomes	Human bone-marrow derived mesenchymal stem cells		Promotion of upregulation of IL-10 and TGF-β1 from PBMCs, stimulation of proliferation and immune-suppression capacity of Tregs in asthmatic patient	[100]
Exosomes	Human umbilical cord mesenchymal stem cells		Amelioration of liver function and restoration of liver fibrosis	[101]
CCR2 positive exosomes	Bone marrow mouse mesenchymal stem cells	C-C motif chemokine receptor-2 (CCR2)	Establishment of protective effects on renal ischemia/reperfusion injury in mouse	[102]
Exosomes	Bone marrow mesenchymal stem cells from rats		Attenuation of inflammation, glial scar formation and of neuronal cells apoptosis, lesion size reduction, improving of axonal regeneration, and of functional recovery after traumatic Spinal Cord Injury in rat model	[103]
Exosomes	Human umbilical cord mesenchymal stem cells		Stimulation of spinal cord injury healing via mitigating the inflammation at the injured area	[104]
